# The Effect of Cream and Gel Vehicles on the Percutaneous Absorption and Skin Retention of a New Eugenol Derivative With Antioxidant Activity

**DOI:** 10.3389/fphar.2021.658381

**Published:** 2021-06-25

**Authors:** Edyta Makuch, Anna Nowak, Andrzej Günther, Robert Pełech, Łukasz Kucharski, Wiktoria Duchnik, Adam Klimowicz

**Affiliations:** ^1^ Department of Chemical Organic Technology and Polymeric Materials, Faculty of Chemical Technology and Engineering, West Pomeranian University of Technology, Szczecin, Poland; ^2^ Department of Cosmetic and Pharmaceutical Chemistry, Pomeranian Medical University in Szczecin, Szczecin, Poland

**Keywords:** bioactive substances, clove water, skin retention, antioxidant activity, vehicles containing new eugenol derivative

## Abstract

The effect of cream and gel vehicles containing clove water on skin permeability was compared for a new eugenol derivative (eugenyl dichloroacetate—EDChA) with antioxidant activity. *In vitro* permeation experiments were conducted in a Franz cell with porcine skin. The cumulative mass and skin accumulation of EDChA were investigated and compared. The antioxidative capacity of the studied vehicles was determined by using the diphenylpicrylhydrazyl (DPPH) free radical reduction method. The antioxidant activity (evaluated with DPPH, ABTS, and the Folin–Ciocalteu methods) of the fluid that penetrated through the pig skin and of the fluid obtained after the skin extraction, were also determined. For comparison, eugenol was also tested. The results of this work could contribute to the development of vehicles with antioxidant potential estimated after 24 h of conducting the experiment, which indicates long-term protection against reactive oxygen species (ROS) in the deeper layers of the skin. The waste water from the clove buds steam distillation -contains several valuable biologically active compounds, and its use is environmentally friendly. We observed that gel vehicles were the best enhancer of skin permeation for both eugenol and its derivative. In most cases, -similar cumulative masses of eugenol and its ester were found in the acceptor fluid. The accumulation of EDChA was higher for cream vehicles in relation to the parent eugenol when applied onto the skin. The greatest amounts of eugenol were accumulated in the skin when these compounds were used in gel vehicles.

## Introduction

Transdermal active substances are a convenient route for administration as they allow minimization of the first-pass metabolism, avoiding gastrointestinal degradation, and providing a controlled and prolonged release of active substances into the systemic circulation. The stratum corneum (SC) protects against external toxins and water loss but also acts as a barrier for the active substance permeation into the skin, which is highly dependent on the lipophilicity and molecular size of these substances. Overcoming the lipophilic barrier, which is the skin, is possible for eugenol and the non-polar, new ester of eugenol, which have molecular weights of <600 Da (Makuch et al., 2020; Ossowicz et al., 2020; Nowak et al., 2021).

Eugenol (4-allyl-2-methoxyphenol) and its ester were derivative characterized by good lipophilicity as determined by the shake-flask method: log *p* eugenol 2.20 ± 0.001, log *p* EDChA 2.65 ± 0.001. Eugenol is a terpene compound classified as an absorption promoter that is characterized by high antibacterial as well as antioxidant activity. Terpenes, which are a group of substances that are commonly considered as safe from the dermal toxicity point of view, are often used in cosmetic vehicles applied to the outer layer of the skin (Makuch et al., 2020). Eugenol has a high potential for application in transdermal systems, it is a cheap and easily available compound that has numerous applications in medicine (Rachoi et al., 2011; Jaganathan et al., 2011; Deepak et al., 2015; Bezerra et al., 2017; Hussain et al., 2011). The antioxidant effect of eugenol and its esters is based on the prevention of free radical formation, oxidative damage repair, and elimination of the damaged particles (Yogalakshmi et al., 2010; Nagababu et al., 2010, Gülçin, 2011; Horvathova et al., 2014; Pérez-Rosés et al., 2016; Bezerra et al., 2017; Janus et al., 2020). Cloves were traditionally used in medical applications, due to their many health benefits, and they are rich in secondary metabolites known for anti-oxidant activity and biological activity (Prashar et al., 2006; Dwivedil et al., 2011; Arung et al., 2011; Legards et al., 2014; Han and Parker, 2017; Pavithra et al., 2019).

Wenkers and Lippold (1999) investigated the skin penetration of 10 nonsteroidal anti-inflammatory drugs (NSAIDs), after the application in a lipophilic vehicle light mineral oil. The results of this research showed that the skin permeability of NSAIDs is a function of the hydrophilicity of the drugs, i.e., of their partition coefficients between phosphate buffer saline (pH 7.4) and the lipophilic vehicle. The skin permeabilities generally increase with increasing hydrophilicity of the NSAIDs. Wenkers and Lippold suggested that the viable *epidermis* provides a decisive barrier to the penetration of NSAIDs from a lipophilic vehicle, based on correlations between skin permeability and octanol–vehicle and PBS–vehicle partition coefficients (Ossowicz et al., 2020; Wenkers and Lippold, 1999).

*In vitro* permeation studies of propranolol hydrochloride were (PH) performed using rat abdominal skin as the permeating membrane in a Franz diffusion cell. The oral bioavailability of PH is poor due to a high first pass metabolism. The patches containing PH of were formulated using a combination of polymers and propylene glycol (polyvinylpyrrolidone, hydroxypropylmethycellulose, and ethyl cellulose) as a plasticizer. The result indicated that the maximum release was obtained with a 2% solution of ethyl cellulose. An optimized batch was evaluated for permeation enhancement through rat skin using the natural permeation enhancer eugenol, and they concluded that permeation enhancement through eugenol was comparable to the commercially available permeation enhancer dimethyl sulfoxide 1%. All the films were found to be stable at 37°C and 45°C with respect to their physical parameters and drug content (Nirav and Rajan, 2011).

There is no doubt that essential oils and their components are able to permeate human skin. But information is rare regarding the percutaneous absorption of essential oils in detail. A study investigated the *in vitro* skin permeation of monoterpenes and phenylpropanoids applied in pure rose oil and in the form of neat single substances. Studies have shown that the application form has an exceeding influence on the skin permeation behavior of the compounds. For substances applied in rose oil, a clear relationship between their lipophilic character, chemical structure, and skin permeation was confirmed. Regarding the P_app_–values, the substances are ranked in the following order: monoterpene hydrocarbons < monoterpene alcohols < monoterpene ketons < phenylpropanoids. In contrast, for neat single substances, there were no relationships between their lipophilic characters, structures, and skin permeation. Except for α-pinene and isomenthone, the P_app_–values of all other substances were several times higher when applied in pure native rose oil compared with their neat form. This suggests that co-operative interactions between essential oil components may promote skin permeation behavior of essential oils and their components (Schmitt et al., 2010).

Oxidative stress is defined as “an imbalance between oxidants and antioxidants in favor of the oxidants, leading to a disruption of redox signaling and control and/or molecular damage” (Sies 2020). Oxidative stress arises when the production of reactive oxygen species overwhelms the intrinsic anti-oxidant defenses (Burton and Jauniaux, 2011) and accumulates in the body by endogenous and exogenous mechanisms (García-Sánchez et al.,). In the human body, the oxidative–antioxidative balance is crucial as it maintains the integrity and functionality of the cell membrane (Kim et al., 2020). Reactive oxygen species can cause a lot of potential damage and are continuously produced by the body’s normal use of oxygen, such as in respiration and certain cell mediated immune functions. ROS, which include free radicals such as superoxide anion radicals, hydroxyl radicals (OH^.^), and non-free-radical species, such as hydrogen peroxide (H_2_O_2_) and singlet oxygen (^1^O_2_), are various forms of activated oxygen [Gulcin et al., 2012]. It is widely recognized that reactive oxygen species contribute to the aging of the skin, the outer barrier of our body, any tissues inside our organism could also be exposed to ROS, both endogenous and exogenous. These compounds, which cause oxidative stress, are responsible for oxidative modifications of polyunsaturated fatty acids and nucleic acids (and as a consequence, for structural changes in cell membranes and DNA damage) (Rincheval et al., 2012; Pisoschi and Pop, 2015; Dam et al., 2019; Wadhwa et al., 2019; Liguori et al., 2018).

In our previous research, we presented the eugenol derivative (eugenyl dichloroacetate—EDChA) made by eugenol esterification with dichloroacetic acid, that can permeate through porcine skin from ethanol (Makuch et al., 2020). Ethanol is a promoters of trans epidermal transport, which has an effect on the effectiveness of the penetration of eugenol and EDChA into the skin. This solvent was able to reversibly transform the structure of the laminar system of the lipid matrix of the *epidermis*, and thus they facilitated the accelerate the diffusion of particles by the stratum corneum. In addition, ethanol can disrupt the function of the skin barrier by affecting the cells between the cellular cement. This results in loosening the lipid layer and increasing its fluidity and, consequently, increases the degree of diffusion of these compounds (Llewelyn et al., 2019; Ossowicz et al., 2020; Nowak et al., 2021). The selectivity of the conversion to EDChA as well as the conversion of eugenol were determined using gas chromatography (GC), while the molar mass of the obtained product was con-firmed based on the mass spectrum (GC-MS). The most important band associated with the presence of an ester group in the structure of the obtained ester was identified using infrared spectroscopy. The unequivocal structure of the new eugenol ester derivative was confirmed with NMR. The antioxidative activity of eugenol and its ester was evaluated by the spectrophotometric method, whereas the values of the n-octanol/water partition coefficient (P) were used to evaluate the lipophilicity (Makuch et al., 2020).

In this study, we compared the effect of a cream and gel, as vehicles, on the skin permeation of eugenol and the new eugenol ester derivative (EDChA). The reason for the cream and gel vehicle application in this study was to evaluate these vehicles on the permeability of the eugenol and EDChA. The results of this work can contribute to the acquisition of knowledge regarding vehicles with antioxidant potential, emphasizing that the water phases are waste from the process of cloves steam distillation and are not reused. The ecological aspect of our research also has importance. The use of waste water from the clove bud steam distillation process is environmentally friendly and allows us to apply the waste, containing valuable biologically active compounds. These compounds, due to their mechanism of action, can have a beneficial effect on the balance between oxidants and antioxidants in the body, minimizing the effects of oxidative stress (Ivy and Payne, 1991; Dahham et al., 2015; Sarpietro et al., 2015; Giovannini et al., 2019; Razafindrakoto et al., 2020). The use of cream and gel vehicles for this type of research is, therefore, justified; moreover, the pH value of the acceptor phase in permeation *in vitro* tests was set at 7.4 for simulation of the skin surface (Ossowicz et al., 2020; Nowak et al., 2021).

## Materials and Methods

### Chemicals

To prepare the studied vehicles vaseline, and cholesterol (Coel, Cracow), beeswax and hydroxyethylcellulose (supplied by Zrob Sobie Krem distributor), clove buds of *Syzygium aromaticum* from Madagascar and Indonesia (Bolinero, Prymat), eugenol p. a. (Keten), distilled water were applied.

To determine of antioxidant activity and skin permeation of active components of prepared formulations (eugenol, EDChA, clove water), 2,2-diphenyl-1-picrylhydrazyl (DPPH), 6-hydroxy-2,5,7,8-tetramethylchroman-2-carboxylic acid (trolox) were purchased from Sigma-Aldrich (United States), ethanol (96% v/v), n-hexane, acetone, methanol, sodium chloride, potassium chloride (all of analytical grade) were from Chempur (Poland) and acetonitrile for HPLC was purchased from J.T. Baker.

### Steam Distillation of Plant Materials and Identification of Water Fractions Obtained During the Steam Distillation by Gas Chromatography-Mass Spectrum Method

First of all, steam distillation of cloves (originating in Madagascar and Indonesia) was carried out with the use of Deryng apparatus. A glass flask with a capacity of 1,000 cm^3^ was filled with 100 g of suitable plant material and 675 g of distilled water, and then the Deryng apparatus was applied to the glass flask. The content of the distillation flask was kept boiling. The process of distillation of plant raw materials was carried out for 5 h, and after the end of the process, the condensate collected in the receivers was separated (using a separator) to obtain the upper aqueous fractions (the clove waters obtained after steam distillation of cloves from Indonesia (water I) and Madagascar (water M).

To identify the substances contained in aqueous fractions, first of all, the upper fractions from the separatory funnel or separator divider were washed in 20 cm^3^ n-hexane. Then the obtained samples were analyzed by gas chromatography coupled with mass spectrometry (GC-MS). Analyses were carried out with the TRACE GC series with the VOYAGER mass detector with a DB5 chromatographic column 30 m long, 0.25 μm in diameter and 0.5 μm film thickness of the stationary phase, using helium as a carrier gas with a flow rate of 1.0 ml/min. The temperature of the dispenser was 240°C, while the volume of the dosed sample was 1 µl. The following temperature gradient was used: 50°C for 1 min, followed by a temperature rise of 8°C/min to 260°C for 5 min, followed by cooling to 50°C.

The qualitative analysis was conducted based on MS spectra. The percentage of a particular compound was determined on the assumption that the sum of all identified compounds is 100%.

### In Vitro Measurement of the Antioxidant Capacity of Clove Water, Eugenol and Its New Ester

Antioxidative activity of ethanolic solutions of eugenol and its new derivative (EDChA) were determined using spectrophotometric method based on DPPH radical reduction as described elsewhere (Brand-Williams et al., 1995; Makuch et al., 2020). The absorbance at the wavelength of 517 nm was measured using Spectroquant Pharo 300 (Merck, Germany). The antioxidant activity of eugenol and its ester was measured as follows: to 2,850 µl of DPPH ethanolic solution (absorbance at 517 nm 1.00 ± 0.02) 150 µl of the sample (containing one of the tested compound at a concentration of 1.000 w/w) was added. The concentration of the analyzed sample in DPPH ethanolic solution was 0.050% w/w. The tube was wrapped in aluminum foil, sealed with a stopper and incubated for 10 min at room temperature. Each sample was prepared in triplicate. After incubation, spectrophotometric measurements were carried out at 517 nm. Solvent applied to obtain extracts was used as reference. The results were expressed as radical scavenging activity (RSA) (Nowak et al., 2019). For each studied compound calibration curve of RSA vs. concentration (0.006–50.000% w/v) was prepared to calculate IC_50_, i.e. the concentration of the compound reducing 50% of free radicals. The concentration range of the analyzed samples in DPPH ethanolic solution were 0.0003–2.5000% w/v.

Moreover, antioxidant activity of water I and M obtained after steam distillation (which was tested unchanged) was evaluated by DPPH method after 10–60 min of incubation (Brand-Williams et al., 1995; Makuch et al., 2020).

### Preparation of Vehicles Containing of Eugenol and Eugenyl Dichloroacetate

To prepare cream vehicles beeswax (0.032 g), cholesterol (0.176 g) and vaseline (3.647 g) were put to glass beaker. The beaker was placed in water bath (70°C) to dissolve the contents. To the second beaker distilled water (5.882 g) and the appropriate amount of either eugenol or its ester were added and mixed using the recipe mixer at 1,375 rpm (Eprus^®^ U500U) to achieve a uniform consistency. Eugenol or its ester were added in an amount of 0.100 g to the aqueous phase of the cream, to obtain the concentration of these compounds (in cream vehicles) of 1.000% w/w. In the next stage, the content of the second beaker was added to the first beaker, and mixed using the recipe mixer to achieve a uniform consistency of the emulsion. The emulsion obtained is water in oil type.

Moreover, for comparison purpose, cream vehicles without active substance were prepared, into which either eugenol or EDChA at a concentration of 1.000% w/w, was entered manually.

Gel vehicles were prepared as follows: hydroxyethylcellulose (0.300 g), distilled water (9.600 g) and the appropriate amount of either eugenol or its ester were added (at a concentration of 1.000% w/w) to glass beaker. The beaker content was mixed manually to achieve a uniform consistency.

In addition, another form (cream and gel vehicles) containing water I and M instead of distilled water was also prepared and evaluated. Cream formulation contained 60.409% w/w of clove water, while gel contained 96.970% w/w of clove water.

### In Vitro Evaluation of Free Radical Scavenging Activity

The antioxidant activity of acceptor fluid taken after 24 h of permeation, and solutions obtained after skin extraction performed after the experiment was determined using the DPPH (according to the procedure described above) (Brand-Williams et al., 1995; Makuch et al., 2020), ABTS (Makuch et al., 2020) and Folin–Ciocalteau (Nowak et al., 2019; Makuch et al., 2020) methods. The ABTS assay is based on the generation of a blue/green ABTS radical, which is applicable to both hydrophilic and lipophilic antioxidant systems; whereas DPPH assay uses a radical dissolved in organic media and is, therefore, applicable to hydrophobic systems (Molyneux 2004).

### Evaluation of Free Radical Scavenging Activity Using Diphenylpicrylhydrazyl Method

Antioxidant activity of vehicles were evaluated using slightly modified DPPH method. The procedure was as follows: to 2,850 µl of DPPH radical solution in acetone (its absorbance at λ = 517 nm was 1.00 ± 0.02) 150 µl of the acetone solution containing the tested formulation at the concentration of 10.0% w/w (0.100% w/w of active substance), while the concentration of active substance in DPPH acetone solution was 0.005% w/w. The tube was wrapped in aluminum foil, sealed with a stopper and then incubated for 10 and up to 60 min at room temperature. Each samples was prepared in triplicate. After incubation, spectrophotometric measurements were carried out at the above mentioned wavelength. The antioxidant activity obtained by this method are expressed in mmol TE/dm^3^, because trolox (TE) was used as a reference substance in the DPPH method. Antioxidant activity of the tested samples was calculated according to the following formula:

$$\text{\%RSA }=\left[\left({\text{A}}_{0}-{\text{ A}}_{p}\right){\text{/A}}_{0}\right]\text{ * }100\text{\% }=\;\left(1\;-{\text{A}}_{p}{\text{/A}}_{0}\right)\text{ * }100\text{\%,}$$

where: %RSA - antioxidant activity, A_0_ - mean value of the absorbance of the acetone solution of DPPH containing 150 µl acetone, A_p_ - mean value of absorbance of the acetone solution of the DPPH radical containing 150 µl of the acetone solution of the tested formulation.

### Evaluation of Free Radical Scavenging Activity Using ABTS Method

First, an aqueous solution of potassium persulfate (2.45 mM) was prepared, to which an appropriate amount of ABTS reagent was introduced to obtain a 7 mM solution of ABTS in an aqueous solution of potassium persulfate. The solution prepared in this way was incubated at 4°C for 24 h and then diluted with methanol (50% v/v) to obtain an absorbance of 1.000 ± 0.020 at 734 nm. The antioxidant activity of acceptor fluid and solutions obtained after skin extraction was measured as follows: 2,500 µl of working ABTS solution and 25 µl of ethanolic solution of tested antioxidant were mixed in spectrophotometric cuvette. The samples prepared in triplicate were incubated for 6 min at room temperature. After this time, the absorbance at 734 nm was measured.

### Evaluation of Total Polyphenole Content Using Folin–Ciocalteu Method

To determine the total content of phenolic compounds in the tested samples the method based on the use of the Folin–Ciocalteu reagent in alkaline medium was applied. The reaction is based on the spectrophotometrically recorded color change of the test solution from yellow to blue. Folin–Ciocalteu reagent was diluted tenfold with water in a dark bottle and incubated at room temperature for 60 min. The antioxidant activity of acceptor fluid and solutions obtained after skin extraction was measured as follows: 1,350 µl of distilled water and 1,350 µl of sodium carbonate solution (0.01 mol/dm^3^) were mixed in spectrophotometric cuvette with 150 µl of the diluted Folin–Ciocalteu solution and 150 µl of an ethanol solution containing the tested samples. The cuvette was sealed with a stopper and incubated for 15 min at room temperature. All the samples were prepared in triplicate. After this time, spectrophotometric measurements were carried out at 750 nm using water as a reference.

### Skin Permeation Studies of Vehicles

The skin permeability for vehicles containing eugenol and EDChA were assessed in a Franz diffusion cell consisted of a 2 ml donor chamber and an 8 ml acceptor chamber. The permeation area was 1 cm^2^. The acceptor fluid, mixed with a magnetic stirrer, was a PBS (phosphate-buffered saline, pH 7.4) solution that maintained the physiological pH. The acceptor chamber was kept at a constant temperature of 37 ± 0.5°C with the VEB MLW Prüfgeräte-Werk type 3,280 thermostat. Before starting the test, Franz diffusion cells were allowed to equilibrate at 37°C for 15 min. Porcine skin was used for the study due to its similar permeability properties to human skin. The skin was from a local slaughterhouse. A fresh portion of skin from the abdomen was washed several times with a solution of PBS. Skin with a thickness of 0.5 mm was cut with a dermatome, and then it was wrapped in aluminum foil and frozen at –20°C for a maximum of 3 months. This freezing time ensured the stability of the skin barrier properties (Zhang et al., 2013). Before the study, the skin was thawed at room temperature for about 30 min, and then it was soaked in a PBS solution for 15 min to hydrate it. In the next stage, the skin was mounted in Franz diffusion cells. The integrity of skin was checked 1 h after its installation in the Franz diffusion chamber (SES GmbH Analyze Systeme, Germany). For this purpose skin impedance was measured using an LCR 4080 m (Conrad Electronic, Germany) operating in parallel mode at 120 Hz (kΩ error <0.5%). To make the measurement, the tips of the probes were immersed in the donor and acceptor chambers filled with the PBS solution. Membranes with an electrical resistance of >3 kΩ, corresponding to the resistance measured for normal human skin, were used in the study (Makuch et al., 2020; Janus et al., 2020).

Preparations (1.000 g) containing one of the test compound (eugenol and EDChA) were placed in the donor chamber. All donor chambers were closed with a plastic stopper to prevent excessive evaporation of the vehicle. The described tests were carried out up to 24 h. An aliquot of 0.3 ml of the solution in the acceptor chamber was taken at specified intervals (30 min, 1, 2, 3, 4, 5, 8, and 24 h), and then supplemented with a fresh portion of buffer of the same pH (Makuch et al., 2020). The samples were analyzed by high-performance liquid chromatography (HPLC).

After completion of permeation experiment, the skin was extracted to estimate the residual amount of tested active ingredients accumulated in it. The antioxidant activity of the obtained extracts was also tested using previously described methods (Brand-Williams et al., 1995). Extraction was carried out as follows: after the experiment was completed, the Franz diffusion chambers were dismantled, while the skin surface was washed three times with an aqueous solution of sodium lauryl sulfate (at a concentration of 0.5% w/w) to rinse of the excess of vehicle containing test compound. A patch (1 cm^2^ diffusion surface) was cut from the skin prepared in this way, dried at room temperature, and then weighed and cut into smaller pieces. Then, 2 ml of concentrated methanol was added, and extraction was carried out for 24 h at 4°C. After 24 h of incubation, the skin was homogenized (for 3 min) using a homogenizer (IKA^®^T18 digital ULTRA TURRAX, Germany). The obtained extracts were then centrifuged at 3,500 rpm for 5 min. The supernatant was analyzed by HPLC to determine the content of active ingredients, while tests on the antioxidant activity of the obtained extracts was evaluated using the DPPH, Folin–Ciocalteau, and ABTS methods.

The cumulative mass of active substance (µg) permeating into the receptor chamber was calculated based on the concentrations of compounds in receptor fluid determined by HPLC. The permeation rate was determined based on the amount of permeated compound over a given period (μg/cm^2^/h). The accumulation of compounds in the skin was calculated by applying the amount of compound obtained after skin extraction; the results are given in μg/cm^2^ of skin (Makuch et al., 2020).

### High-Performance Liquid Chromatography Analysis

The samples were analyzed by high-performance liquid chromatography (HPLC) with a UV detector (Knauer, Berlin, Germany). The components tested were separated on a 125 × 4 mm column containing Hyperisil ODS; particle size 5 µm. The flow rate of the mobile phase, consisted of acetonitrile, water, and MeOH (28:64:8, by vol), was 1 ml/min. Twenty microliters of each analyzed sample was injected onto the column.

### Statistical Analysis

Statistical calculations were done using Statistica 13 PL software (StatSoft, Polska). The results were evaluated using one-way analysis of variance (ANOVA). Significant differences were evaluated using Tukey post-hoc test. Probabilities *p* < 0.05 were considered to be statistically significant. Results are presented as the mean ± standard deviation (SD).

## Results

**FIGURE 1:**
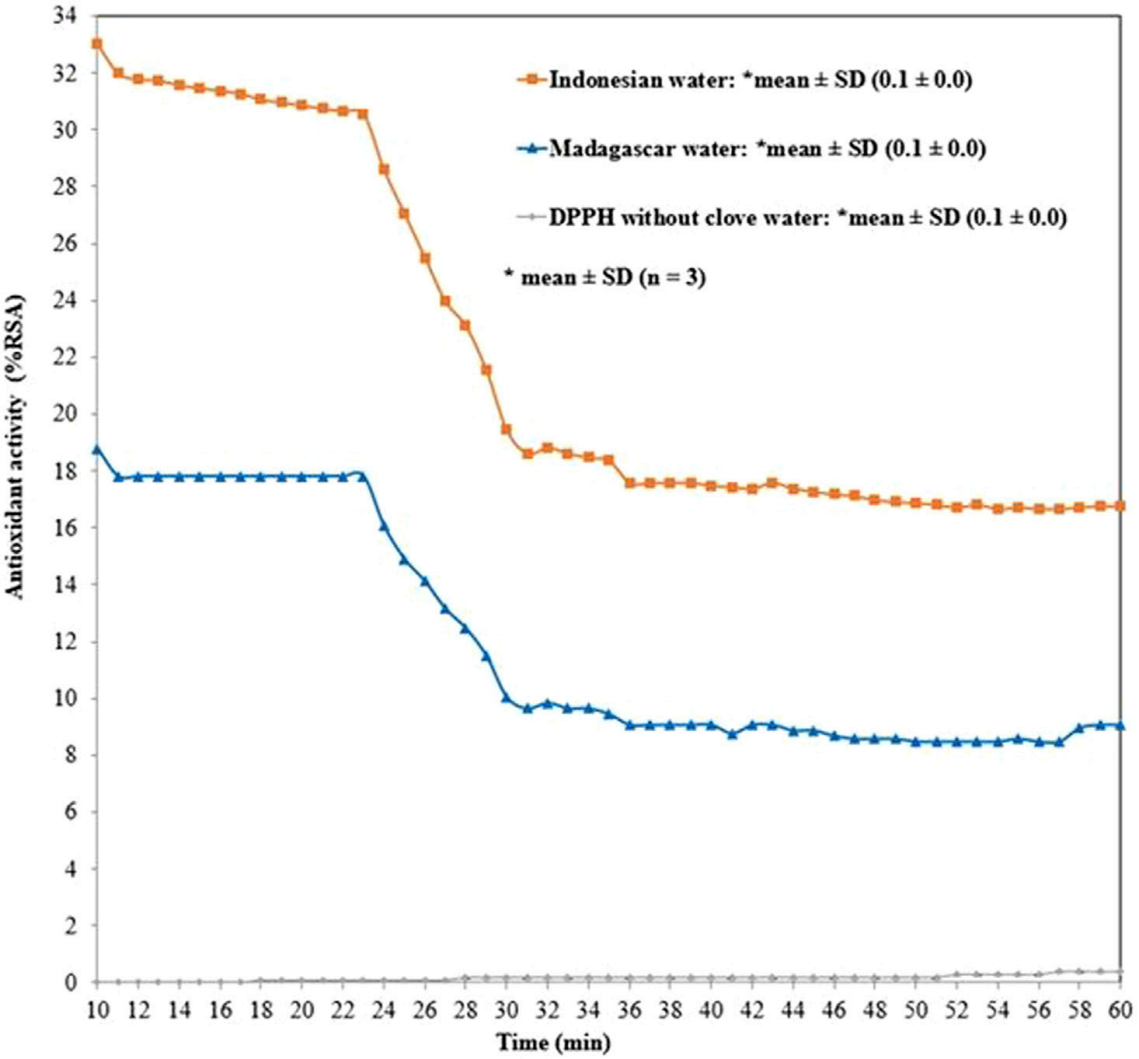
Time course evolution of the antioxidant activity of the clove water evaluated with DPPH method: hexane extract of water I containing: furfural 0.98%, benzyl alcohol 0.32%, methyl salicylate 0.27%, 4-allilofenol 0.41%, eugenol 94.45%, β-caryophyllene 1.21%, α-caryophyllene 0.20%, eugenyl acetate 2.14%; hexane extract of water M containing: furfural 0.13%, benzyl alcohol 0.05%, methyl salicylate 0.29%, 4-allilofenol 0.22%, eugenol 89.21%, β-caryophyllene 7.61%, α-caryophyllene 1.27%, eugenyl acetate 0.87%, β-caryophyllene oxide 0.35%.

Table 1 presents antioxidant activity of eugenol and its ester derivative, carried out by the DPPH method.Figure 1 presents the antioxidant activity of the clove water obtained after steam distillation of cloves from Indonesia (water I) and Madagascar (water M).

**TABLE 1:** Concentration of eugenol and EDChA reducing 50% of free radicals.

IC_50_ (µM)	Antioxidant activity (DPPH method)	
	Eugenol	EDChA
	%RSA	
	*6.1	**4,276.0

The studied compounds showed different antioxidant activity determined by DPPH method - Table 1. Studies have shown that the values of the parameter determining the concentration reducing 50% of free radicals (IC_50_) for eugenol are inversely proportional to its antioxidant activity, i.e. the lower the IC_50_ the higher antioxidant activity. Eugenol (IC_50_ = 6.1 µM) had the highest activity. The value of the IC_50_ parameter for eugenol was more than 8 times lower than the value described in the literature for this compound (IC_50_ = 50.44 µM) (Floegel et al., 2011).

The qualitative analysis carried out on the vehicles of MS spectra showed that eight main compounds were identified in water M: furfural 0.98%, benzyl alcohol 0.32%, methyl salicylate 0.27%, 4-allylphenol 0.41%, eugenol 94.45%, β-caryophyllene 1.21%, α-caryophyllene 0.20%, eugenyl acetate 2.14%. The following compounds were identified in water I: furfural 0.13%, benzyl alcohol 0.05%, methyl salicylate 0.29%, 4-allylphenol 0.22%, eugenol 89.21%, β-caryophyllene 7.61%, α-caryophyllene 1.27%, eugenyl acetate 0.87%, β-caryophyllene oxide 0.35%. The antioxidant activity along with the prolongation of the reaction time between DPPH radical and antioxidant decreased from 33.0 ± 0.0 %RSA (at 10 min) to 16.8 ± 0.1 %RSA (at 60 min, in the case of water I) and from 18.8 ± 0.1 %RSA (at 10 min) to 9.1 ± 0.1 %RSA (at 60 min, in the case of water M)–Figure 1.

Table 2 presents the results for the antioxidant activity of formulations containing of eugenol and EDChA, carried out by the DPPH method.

**TABLE 2:** Antioxidant activity of vehicles.

Sample number	Cream vehicles containing	^a^Antioxidant activity (DPPH method)	
		% RSA_10_	% RSA_60_
1	Pure with distilled water	n.a	n.a
2	^b^Pure with water M	8.2 ± 0.1 k	9.9 ± 0.1 m
3	^b^Eugenol	12.2 ± 0.1 h	38.0 ± 0.1g
4	^b^ ^,^ ^c^EDChA	16.6 ± 0.1 f	38.0 ± 0.1 g
5	^b^ ^,^ ^d^Eugenol	10.9 ± 0.1 i	23.4 ± 0.1 j
6	^b^ ^,^ ^d^EDChA	16.2 ± 0.1 g	33.2 ± 0.1 h
7	^b^ ^,^ ^e^Eugenol	10.7 ± 0.1 i	20.9 ± 0.1 k
8	^b^ ^,^ ^e^EDChA	17.0 ± 0.1 f	24.3 ± 0.0 i
9	^b^ ^,^ ^f^Eugenol	22.5 ± 0.1 d	59.6 ± 0.1 c
10	^b^ ^,^ ^f^EDChA	20.8 ± 0.1 e	41.5 ± 0.1 e
11	^g^Pure with water I	16.4 ± 0.1 g	18.2 ± 0.1 l
12	^f^ ^,^ ^g^Eugenol	31.5 ± 0.1 b	65.5 ± 0.1 b
13	^f^ ^,^ ^g^EDChA	25.3 ± 0.1 c	49.5 ± 0.1 d

Sample number	Gel vehicles containing	*Antioxidant activity (DPPH method)	
		% RSA_10_	% RSA_60_
14	Pure with distilled water	n.a	n.a
15	^b^Eugenol	78.6 ± 0.2 a	90.0 ± 0.2 a
16	^b^EDChA	68.7 ± 0.1 b	89.2 ± 1.4 a

a Mean ± SD (*n* = 3).

b Water M, which is a waste from the steam distillation of cloves from Madagascar.

g Water I, which is a waste from the steam distillation of cloves from Indonesia.

c The vehicle was first obtained and then the relevant amount of active substance was entered with a recipe mixer.

d The relevant active substance was added to the organic phase during the preparation of the vehicle.

e The relevant active substance was added to the aqueous phase during the preparation of the vehicle.

f The vehicle was first obtained and then the relevant active substance was (manually) screwed into it.

n.a. - no activity.

a-m - values are significantly different between analyzed vehicles (*p* = 0.001).

% RSA10 - The antioxidant activity of the analysed sample was measured after 10 minutes incubation at room temperature.

% RSA60 - The antioxidant activity of the analysed sample was measured after 60 minutes incubation at room temperature.

Tables 3 and 4 presents the results for the antioxidant activity of solutions of the tested vehicles containing of eugenol and EDChA.

**TABLE 3:** Antioxidant activity of solutions of cream vehicles evaluated with DPPH, ABTS and Folin-Ciocalteu methods.

Sample number	Cream vehicles containing	Vehicles applied to the skin	Acceptor fluid after 24 h of permeation	Solution after skin extraction
		^a^Antioxidant activity (DPPH method): mmol TE/dm^3^ (% RSA)		
**1**	Pure with distilled water	n.a	n.a	n.a
**2**	^b^Pure with water M	(8.2 ± 0.1 i)	<0.1 (<0.8)	<0.1 (<0.5)
**3**	^b^ ^,^ ^c^Eugenol	(12.2 ± 0.1 g)		
**4**	^b^ ^,^ ^c^EDChA	(16.6 ± 0.1 e)		
**5**	^b^ ^,^ ^d^Eugenol	(10.9 ± 0.1 h)		
**6**	^b^ ^,^ ^d^EDChA	(16.2 ± 0.1 f)		
**9**	^b^ ^,^ ^f^Eugenol	(8.2 ± 0.1 i)		
**10**	^b^ ^,^ ^f^EDChA	(15.4 ± 0.1 h)		
**11**	^g^Pure with water I	(22.5 ± 0.1 d)		
**12**	^f^ ^,^ ^g^Eugenol	(17.1 ± 0.1 e)		
**13**	^f^ ^,^ ^g^EDChA	(16.4 ± 0.1 f)		
**7**	^b^ ^,^ ^e^Eugenol	0.1 ± 0.1 c (10.7 ± 0.1 c)	0.1 ± 0.1 c (0.8 ± 0.2 c)	0.1 ± 0.1 b (4.0 ± 0.3 b)
**8**	^c^ ^,^ ^b^EDChA	0.1 ± 0.1 a (17.0 ± 0.1 a)	0.1 ± 0.1 a (1.6 ± 0.5 a)	0.1 ± 0.1 ab (5.3 ± 0.6 ab)
		**^a^Antioxidant activity (ABTS method): Mmol TE/dm^3^ (% RSA)**		
**1**	Pure with distilled water	—	n.a	n.a
**2**	^b^Pure with water M		<0.1 (<0.5)	<0.1 (<5.4)
**3**	^b^ ^,^ ^c^Eugenol	—	<0.1 (<4.3)	<0.3 (<16.2)
**5**	^b^ ^,^ ^d^Eugenol			
**9**	^b^ ^,^ ^f^Eugenol			
**12**	^f^ ^,^ ^g^Eugenol			
**4**	^b^ ^,^ ^c^EDChA	—	<0.1 (<3.8)	<0.3 (<15.6)
**6**	b ^,^ ^d^EDChA			
**10**	^b^ ^,^ ^f^EDChA			
**13**	^f^ ^,^ ^g^EDChA			
**11**	^g^Pure with water I	—	<0.1 (<2.2)	<0.3 (<14.6)
**7**	^b^ ^,^ ^e^Eugenol	—	0.1 ± 0.1 a (8.8 ± 0.5 a)	0.4 ± 0.1 a (20.3 ± 0.6 a)
**8**	^b^ ^,^ eEDChA	—	0.1 ± 0.1 b (7.6 ± 0.5 b)	0.4 ± 0.1 a (19.9 ± 0.6 a)
		**^a^Total polyphenole content (folin-ciocalteu method) mmol GA/dm^3^**		
**1**	Pure with distilled water	—	n.a	n.a
**2**	^b^Pure with water M	—	<0.1	<0.2
**3**	^b^ ^,^ ^c^Eugenol			
**4**	^b^ ^,^ ^c^EDChA			
**5**	^b^ ^,^ ^d^Eugenol			
**6**	^b^ ^,^ ^d^EDChA			
**9**	^b^ ^,^ ^f^Eugenol			
**10**	^b^ ^,^ ^f^EDChA			
**12**	^f^ ^,^ ^g^Eugenol			
**13**	^f^ ^,^ ^g^EDChA			
**15**	^g^Pure with water I			
**7**	^b^ ^,^ ^e^Eugenol	—	0.1 ± 0.1 a	0.2 ± 0.1 a
**8**	^b^ ^,^ ^e^EDChA			0.3 ± 0.1 a

a Mean ± SD (*n* = 3).

b water M, which is a waste from the steam distillation of cloves from Madagascar.

g water I, which is a waste from the steam distillation of cloves from Indonesia.

c The vehicle was first obtained and then the relevant active substance was (with a recipe mixer) screwed into it.

d The relevant active substance was added to the organic phase during the preparation of the vehicle.

e The relevant active substance was added to the aqueous phase during the preparation of the vehicle.

f The vehicle was first obtained and then the relevant active substance was (manually) screwed into it.

n.a. - no activity.

a-i - values are significantly different between analyzed substances (*p* = 0.001).

**TABLE 4:** Antioxidant activity of solutions of gel vehicles evaluated with DPPH, ABTS and Folin-Ciocalteu methods.

Sample number	Gel vehicles containing	Vehicles applied to the skin	Acceptor fluid after 24 h of permeation	Solution after skin extraction
		^a^Antioxidant activity (DPPH method): mmol TE/dm^3^ (% RSA)		
**14**	Pure with distilled water	n.a	n.a	n.a
**15**	Eugenol	0.5 ± 0.1 a (78.6 ± 0.2 a)	0.1 ± 0.1 b (7.6 ± 0.5 b)	0.2 ± 0.1 b (32.4 ± 0.3 b)
**16**	EDChA	0.4 ± 0.1 b (68.7 ± 0.1 b)	0.1 ± 0.1 a (12.3 ± 0.2 a)	0.3 ± 0.1 a (45.2 ± 0.6 a)
		**^a^Antioxidant activity (ABTS method): Mmol TE/dm^3^ (% RSA)**		
**14**	Pure with distilled water	—	n.a	n.a
**15**	Eugenol	—	0.4 ± 0.1 a (22.3 ± 0.5 a)	1.2 ± 0.1 a (79.7 ± 0.6 a)
**16**	EDChA	—	0.8 ± 0.1 b (36.4 ± 0.4 b)	2.0 ± 0.1 a (84.7 ± 0.4 a)
		**^a^Total polyphenole content (folin-ciocalteu method): Mmol GA/dm^3^**		
**14**	Pure with distilled water	—	n.a	n.a
**15**	Eugenol	—	0.2 ± 0.1 a	0.5 ± 0.1 a
**16**	EDChA	—	0.1 ± 0.1 a	

a Mean ± SD (*n* = 3), n.a. - no activity a-b - values are significantly different between analyzed substances (*p* = 0.001).

Tables 5 presents the results of studies on the permeation and the accumulation of active substances contained in vehicles.

**TABLE 5:** Permeation of active substances (contained in contained in tested vehicles) through skin and the amounts of extracted active ingredients accumulated in it.

Sample number	Cream vehicles containing	Cumulative mass of substance after 24 h of permeation test: (µg)	Skin accumulation of substance: (µg/cm^2^ skin)
**7**	^a^Eugenol	20.5 ± 0.8 a	156.9 ± 7.0 b
**8**	^a^EDChA	19.6 ± 1.8 a	173.9 ± 8.4 b

Sample number	Gel vehicles containing	Mass of substance in the acceptor fluid after 24 h of permeation: (µg)	Skin accumulation of substance: (µg/cm^2^ skin)
**15**	^a^Eugenol	31.3 ± 4.3 a	334.4 ± 20.4 b
**16**	^a^EDChA	28.8 ± 2.6 b	255.9 ± 20.1 c

a Mean ± SD (*n* = 3), a-c - values are significantly different, mass substance in the acceptor fluid and concentration of substance extracted from the skin (*p* = 0.001).

Cumulative mass of substance after 24 h of permeation test (μg): The cumulative mass of active substance (μg) permeating into the receptor chamber. Mass of substance in the acceptor fluid after 24 h of permeation (μg): The amount of permeated compound over a given period (μg). Skin accumulation of substance (μg/cm^2^ skin): The accumulation of compound in the skin, which obtained after skin extraction; and the results are given in μg/cm^2^ of skin.

**FIGURE 2:**
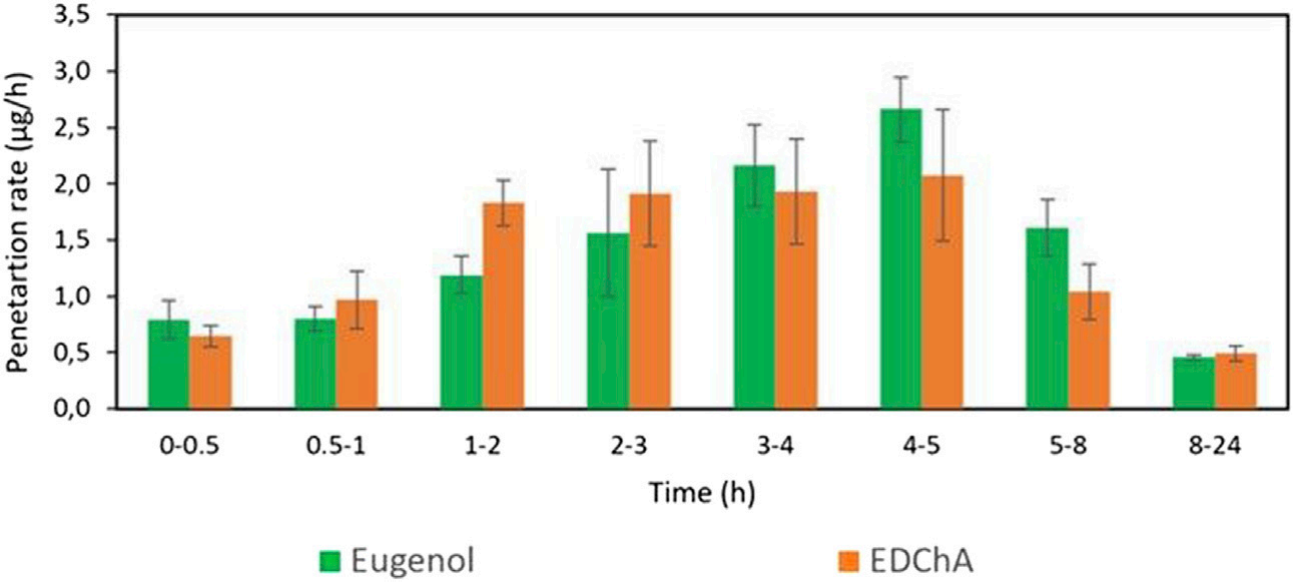
The penetration rate of Eugenol, EDChA contained in cream vehicles through the skin during the 24 h experiment.

**FIGURE 3:**
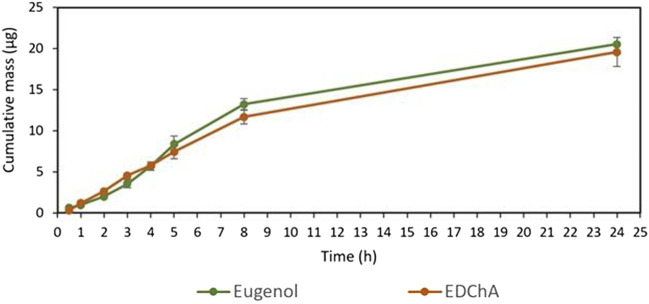
Cumulative mass of Eugenol, EDChA contained in cream vehicles penetrated into acceptor fluid during the 24 h experiment.

Figures 2, 3 show of the comparison of *in vitro* permeation profiles for eugenol and EDChA contained in cream vehicles through the skin during the 24 h experiment.

**FIGURE 4:**
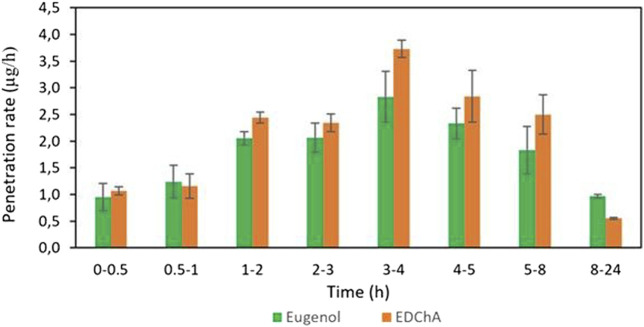
The penetration rate of Eugenol, EDChA contained in gel vehicles through the skin during the 24 h experiment.

**FIGURE 5:**
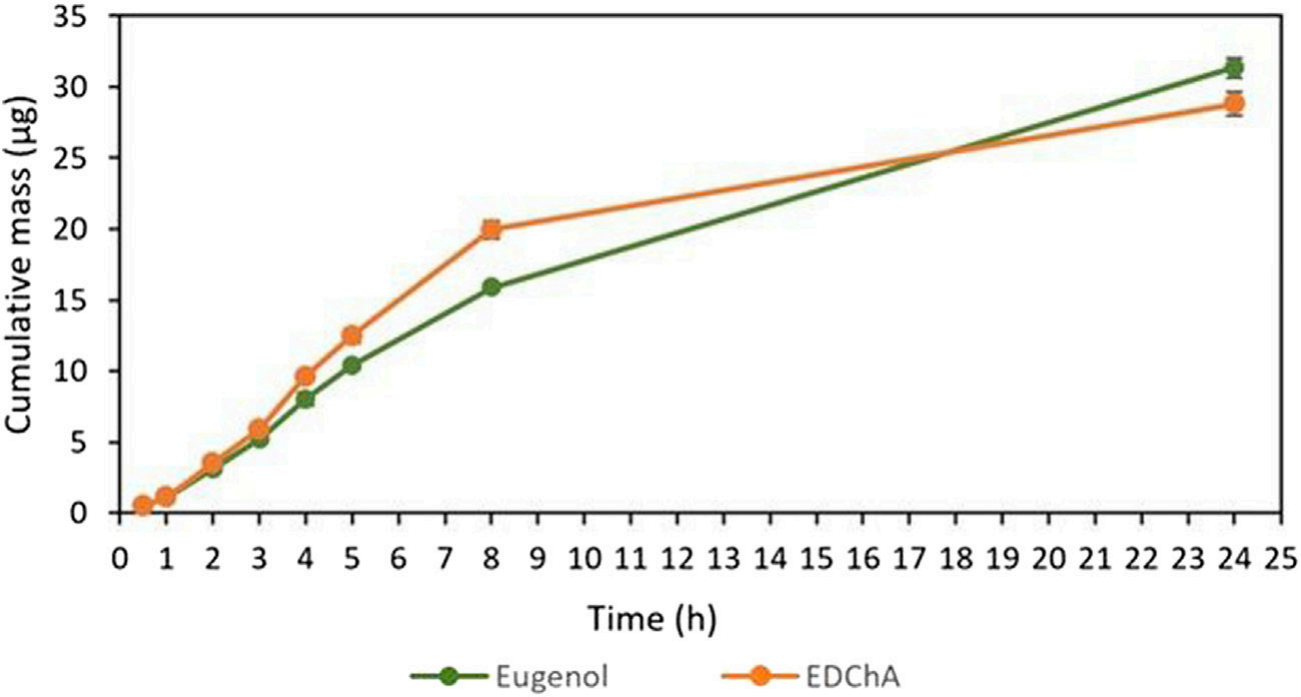
Cumulative mass of Eugenol, EDChA contained in gel vehicles penetrated into acceptor fluid during the 24 h experiment.

Figures 4, 5 show of the comparison of *in vitro* permeation profiles for eugenol and EDChA contained in gel vehicles through the skin during the 24 h experimen

The study of DPPH radical scavenging capacity of the pure vehicles, containing no active substance (sample 1 and 14) and the vehicle prepared with the use of clove water as post-processing waste (sample two and sample 11) showed that sample one did not show antioxidant activity, while samples 2 and 11 were characterized by a DPPH radical scavenging degree of: 8.2 ± 0.1% RSA_10_ and 9.9 ± 0.1% RSA_60_ and 16.4 ± 0.1% RSA_10_ and 18.2 ± 0.1% RSA_60_, respectively. Cream vehicle containing 1.000% w/w antioxidant (eugenol and EDChA) were characterized by the capacity to react with DPPH radical. The highest efficacy was shown by the vehicles obtained in the following way: first a vehicle containing clove water was obtained, and then a suitable active substance (i.e. eugenol, a new eugenol ester derivative - EDChA) was added (manually) into the final vehicle. The vehicles showed the highest efficiency to react with DPPH radical after 60 min of incubation. %RSA of these samples decreased as follows: sample 12 (65.5 ± 0.1) > sample 9 (59.6 ± 0.1) > sample 13 (49.5 ± 0.1)–Table 2.

Additionally, gel vehicle containing 1.000% w/w eugenol (samples 15) and EDChA (samples 16) were characterized by efficacy to react with DPPH radical. Samples 15 and 16 showed the highest efficiency to react with DPPH radical after 60 min of incubation. %RSA for these samples was respectively: 90.0 ± 0.2 (sample 15) and 89.2 ± 1.4 (sample 16)–Table 2.

In the case of studies carried out for pure vehicle with distilled water instead of antioxidant solution (sample 1 - Table 3, sample 14 - Table 4), no antioxidant activity was shown (vehicle applied to the skin, acceptor fluid after 24 h of permeation, solution after skin extraction). The test results, presented in Table 3, show that solutions of acceptor fluids containing eugenol and EDChA were characterized by antioxidant activity evaluated with DPPH, ABTS and Folin-Ciocalteu methods. The degree of reduction of the DPPH free radical (of acceptor fluid collected after 24 h of permeation) decreased in the following order: 1.6% RSA (for vehicle containing EDChA - sample 8) > 0.8% RSA (for vehicle containing eugenol - sample 7). Antioxidant activity of acceptor fluids after 24 h of permeation of samples 2–4, six to seven and samples 9–12 were low and were below <0.8% RSA - Table 3. The antioxidant activity (determined by the ABTS method) of acceptor fluid collected after 24 h of permeation showed that the vehicle containing eugenol had the highest antioxidant activity (8.8% RSA). Lower antioxidant activity was observed for the vehicle with EDChA (7.6% RSA). The lowest antioxidant activity was observed for the samples: 2 (<0.5% RSA), and 11 (<2.2% RSA), 4, 6, 10 and 13 (<3.8% RSA) and 3, 5, 9 and 12 (<4.3% RSA) - Table 3. Acceptor fluid collected during 24 h of penetration of the tested vehicles containing EDChA and eugenol were characterized by the highest polyphenol content (0.1 ± 0.1 mmol GA/dm^3^). In contrary, the lowest concentrations were found for samples: 2–6, 9–12 and 13 (<0.1 mmol GA/dm^3^) - Table 3.

The results of studies on the antioxidant activity of solutions of skin extracts obtained after the experiment showed that vehicles containing eugenol (sample 7) and its ester derivative (sample 8) were characterized by antioxidant activity, as estimated by the three techniques applied: DPPH, ABTS, and Folin–Ciocalteu. The degree of DPPH free radical reduction for these vehicle decreased in the following order: EDChA (5.3 ± 0.6) > eugenol (4.0 ± 0.3% RSA). The degree of DPPH free radical reduction for samples: 2–6, 9–13 was below 0.5% RSA - Table 3.

In contrary, the antioxidant activity (ABTS method) of the samples obtained after skin extraction decreased as follows: 20.3 ± 0.6 (sample 7) > 19.9 ± 0.6 (sample 8). The antioxidant activity of the skin: sample two was below 5.4% RSA, sample 11 was below 14.6% RSA, samples 4, 6, 10, and 13 was below 15.6% RSA and samples 3, 5, 9, and 12 was below 16.2% RSA - Table 3.

The results obtained by the Folin–Ciocalteu method showed that the value of antioxidant activity of solution obtained after skin extraction (vehicle containing EDChA) was higher (0.3 ± 0.1 mol GA/dm^3^) than the value of antioxidant activity of vehicle containing eugenol (0.2 ± 0.1 mmol GA/dm^3^). The values of antioxidant activity of solutions obtained after skin extraction of samples 2–6, 9–10, 12–13, and 15 was below 0.2 mmol GA/dm^3^ - Table 3.

The results, presented in Table 4, show that solutions of acceptor fluids containing eugenol and EDChA were characterized by antioxidative activity evaluated with DPPH, ABTS and Folin-Ciocalteu methods. The degree of reduction of the DPPH free radical (of acceptor fluid collected after 24 h of permeation) decreased in the following order: 12.3 ± 0.2 (for gel vehicle containing EDChA - sample 16) > 7.6 ± 0.5% RSA (in the case of gel vehicle containing eugenol–sample 15). The antioxidant activity (determined by the ABTS method) of acceptor fluid collected after 24 h of permeation showed that the vehicle containing EDChA had the highest antioxidant activity (36.4 ± 0.4% RSA). Lower antioxidant activity was observed for the vehicle with eugenol (22.3 ± 0.5% RSA). The results obtained by the Folin–Ciocalteu method showed that the values of antioxidant activity of solutions obtained after skin extraction (sample 15 and 16) were 0.5 ± 0.1 mmol GA/dm^3^ - Table 4.

In our *in vitro* research, the permeation of vehicle containing eugenol and its new ester derivative (EDChA) through pig skin was assessed. The experiment was carried out using a Franz diffusion chamber, in which the donor phase consisted of the vehicles tested. The acceptor phase was PBS solution, because it corresponds to systemic conditions, is isotonic in nature, and resembles conditions prevailing in the deeper layers of the skin (Makuch et al., 2020). As shown in Table 5, the application of ester of eugenol in each of the cream and gel as vehicles not led to increase in the skin permeation of EDChA in comparison to eugenol applied in the same vehicle. After conducting the experiment for 24 h, the highest average cumulative mass was observed in the case of eugenol (contained in cream vehicle 20.5 ± 0.8 µg, contained in gel vehicle 31.3 ± 4.3). The mass was slightly lower in the case of EDChA (in the case of cream vehicle 19.6 ± 1.8 µg, in the case of gel vehicle 28.8 ± 2.6). The highest permeation rate of cream vehicles containing eugenol and EDChA to the acceptor fluid (µg/h) was observed between 4 and 5 h (Figure 2), while for gel vehicles between 3 and 4 h (Figure 4). Moreover, the average cumulative masses for vehicles containing eugenol and EDChA at 0.5; 1; 2; 3; 4; 5; 8, and 24 h are shown in Figure 3 and Figure 5. In our study, differences in permeation of active substances were found, depending on the type of vehicle used (see Table 5). Considering the average cumulative mass of the com-pounds, the permeation from the vehicles used was ranked in the following order: gel vehicles containing eugenol > gel vehicles containing EDChA > cream vehicles containing eugenol > cream vehicles containing EDChA.

After the experiment was carried out, the skin was extracted in order to evaluate the amount of the accumulated tested active ingredients. The obtained results showed that the concentration of substances (contained in the cream vehicles) in the analyzed extracts decreased in the following order: EDChA (173.9 ± 8.4 μg/cm^2^ skin) > eugenol (156.9 ± 7.0 μg/cm^2^ skin). Moreover the concentration of substances (contained in the gel vehicles) in the analyzed extracts decreased in the following order: eugenol (334.4 ± 20.4 μg/cm^2^ skin) > EDChA (255.9 ± 20.1 μg/cm^2^ skin) - Table 5.

## Discussion

Eugenol has a hydroxyl group (-OH) associated with an aromatic ring with acidic properties, which could lead to antioxidant activity. Its free radicals scavenging activity could lead to form phenolic radicals. These radicals are stable due to resonance caused by charge transfer and are not able to detach hydrogen from lipid or protein molecules (and to decrease the oxidation). Replacement of hydrogen atoms in the aliphatic chain EDChA by heteroatoms (in this case, chlorine atoms) enhances the anti-oxidative properties. Eugenol esters containing chlorine atoms in the structure easily trap free radicals, giving up the H atom in the aliphatic chain. The reason is a change in the shape of the molecule, i.e. a change in length, direction, range and polarization of the bonds and a change in the symmetry of the particles. Introduction of chlorine atoms into the structure, causes polarization of bonds between carbon-chlorine atoms. The polarization of bonds between the carbon-chlorine atoms reduces the density of the electron cloud in the whole molecule and causes polarization of all close bonds present in the structure. As a result of this bond between the carbon-hydrogen atoms in EDChA molecules, they change their length and polarity. In addition, the presence of chlorine atoms in the structure of EDChA changes the electro-neutrality of carbon atoms. Moreover, the presence of the methoxy group (-OCH_3_) in the eugenol and its ester increases the antioxidant properties of these compounds (Makuch et al., 2020).

We demonstrated that cosmetic vehicles (both cream and gel) containing eugenol and new eugenol derivatives (EDChA), penetrated through biological membranes. The eugenol derivative (eugenyl dichloroacetate—EDChA) made by eugenol esterification with dichloroacetic acid, had a similar permeation through porcine skin compared to the starting eugenol. This study showed that newly developed eugenol modifications could be promising active ingredients into formulations applied to the skin and employed as an ideal alternative to commercial eugenol. We noticed that the type of vehicles (cream or gel) influenced the eugenol and EDChA transport through porcine skin. We observed that gel vehicles were the best enhancer of the skin permeation of both eugenol and its derivative. In most cases, a similar cumulative mass of eugenol and its ester was found in the acceptor fluid.

Relationship was found between the lipophilicity of eugenol and its ester derivative in cream and gel vehicles and skin accumulation. The accumulation of EDChA was higher for cream vehicles in relation to the parent eugenol applied onto the skin. The greatest amounts of eugenol were accumulated in the skin when these compounds were used in gel vehicles.

A relationship was also found between the antioxidant activity of vehicles containing clove water and vehicles containing distilled water. The highest antioxidant activity determined with the DPPH method was found for gel vehicles containing EDChA and eugenol (Table 2) as active substances and clove water (the aqueous fraction containing furfural, benzyl alcohol, methyl salicylate, 4-allilofenol, eugenol, β-caryophyllene, α-caryophyllene, eugenyl acetate, and β-caryophyllene oxide) as a water phase. These compounds, due to their mechanism of action, can have a beneficial effect on the balance between oxidants and antioxidants in the body, minimizing the effects of oxidative stress. In addition, the good permeability of vehicles containing eugenol and EDChA through the skin and their proper accumulation in the skin (Table 5; Figures 2, 3) as well as their antioxidant capacity (Tables 3, Tables 4) could also reduce the exogenous effects of free radicals. Cloves are rich in secondary metabolites known for antioxidant activity. These preliminary results highlighted, for the first time, that clove water showed antioxidant activity. Thus, these first findings support the use of clove water, in vehicles; however, more studies are needed to better clarify the antioxidant mechanisms. The second direction of research on the proposed vehicles, which should be developed in the near future, is to investigate the correlation between the content of extra compounds next to eugenol or the new eugenol derivative and the antioxidant effect of test vehicles. Perhaps some of these additional compounds (identified in clove water) in combination with eugenol or EDChA will increase the antioxidant properties of the vehicles. Taking into consideration possible future use of the tested vehicles, the continuation of these tests has the potential for further applications.

## Data Availability

The raw data supporting the conclusion of this article will be made available by the authors, without undue reservation.
